# Pancreatic Islet Cell Transplantation: Graft Stability and Metabolic Outcomes

**DOI:** 10.21926/obm.transplant.2003115

**Published:** 2020-07-07

**Authors:** Khawla F. Ali, Betul Hatipoglu

**Affiliations:** 1 Department of Medicine, Royal College of Surgeons in Ireland-Medical University of Bahrain, Bahrain; 2 Endocrinology and Metabolism Institute, Cleveland Clinic, Cleveland, OH, USA

**Keywords:** Islet transplant, beta cells, autologous transplant, allogenic transplant

## Abstract

Pancreatic islet transplantation is a rapidly evolving field. It has been increasingly regarded as a promising approach for the correction of dysglycemia associated with type 1 diabetes mellitus (allogenic islet transplantation), or the prevention of surgical diabetes in chronic pancreatitis subjects undergoing total pancreatectomy (autologous islet transplantation). In this review, we discuss the latest literature pertaining to metabolic outcomes of autologous and allogenic islet transplantation, shedding close light on our own latest experience in the autologous islet transplantation setting.

## 1. Introduction

Islet transplantation has been increasingly recognized as a viable approach for maintenance and/or restoration of beta cell function. Pancreatic islet transplantation is either performed to correct the dysglycemia associated with type 1 diabetes mellitus [1, 2], or ameliorate surgical diabetes in chronic pancreatitis patients undergoing total-pancreatectomy [3, 4]. Restitution of endogenous hormonal regulation of glucose levels via transplantation has been shown to significantly improve metabolic outcomes, reduce progression of diabetes-related complications and improve quality of life [5–7]. In this report, we review the current knowledge on pancreatic islet transplantation, shedding light on the durability and metabolic outcomes of autologous and allogenic islet transplantation.

## 2. Autologous Islet Transplantation

Total pancreatectomy (TP) has been increasingly performed forthe treatment of chronic pancreatitis (CP) refractory to medical therapy [8–10]. In order to ameliorate the surgical dysglycemia associated with post-pancreatectomy states, the procedure is often coupled with isolation of islets of Langerhans from the resected pancreas, and subsequent infusion of islets back into the host’s liver via the portal vein. The surgical procedure, total pancreatectomy and autologous islet transplantation (TPAIT), has been shown to significantly improve pain control, insulin independence rates and overall quality of life in subjects with CP [7, 10].

The glycemic outcomes of TPAIT differ across patients. Previous reports at 28–36 months post-TPAIT demonstrated that a third of transplanted patients become normoglycemic and insulin independent, a third show partial islet graft function requiring some exogenous insulin, and a third become fully dependent on insulin therapy [4, 11]. A critical factor in predicting insulin independence post-TPAIT is the mass of islets transplanted, often expressed as islet equivalent per kg of bodyweight (IEQ/kg). Subjects receiving >5000 IE/kg of bodyweight are at greater odds of achieving insulin independence, whilst those receiving fewer than 2500 IEQ/kg of body weight are more likely to become insulin dependent post-TPAIT [4, 11, 12].

However, up to this point, there had been lack of longitudinal data examining the change in engrafted beta cell function over time. In the past year, our group published the first report on the effects of time-lapse on beta cell functionality post-TPAIT [13]. In our analysis, we observed a steady and significant decline in functional beta cell capacity in the first 2 years following transplantation. A steeper decline in graft function was particularly observed in the first 6 months following TPAIT, with the functional decline persisting, but at a slower rate thereafter [13]. The assessment of beta cell functionality in our analysis, as it relates to time, was done via the novel BETA-2 scoring assessment along with the classic mixed-meal tolerance testing. The former tool being a validated refinement of the classic beta-score, a well-established index of islet graft function [14, 15]. The novel BETA-2 score integrates fasting C-peptide, fasting plasma glucose, hemoglobin A1c (HbA1c), and insulin doses to better detect abnormal glucose tolerance and insulin independence following pancreatic islet transplantation. Originally developed and utilized in the allogenic setting, our study was the first to report its applicability in the autologous islet transplantation setting [13].

The observed early and persistent decline in islet function has been hypothesized to be due to injury of grafted cells during the transplantation process [16–18]. Harmful events are induced in the early posttransplant period by stressors such as hypoxia, hyperglycemia, and the infiltrating innate immune cell-derived cytokines and other pro-inflammatory factors. The isolation procedure itself leads to the devascularization of islets which results in relative underoxygenation, leading eventually to reestablishment of arterialization and adequate oxygenation of intrahepatic islets [19, 20]. Additionally, compelling level of evidence indicts the innate immunity-driven inflammation for the stress and decline in beta cell function in the autologous setting [16–18]. Pro-coagulatory and pro-inflammatory cascades are activated within minutes after islet infusion back into the host and actively contribute to the stress and injury of transplanted islets. The levels of inflammatory cytokines such as IL-1Ra, IL-6, IL-8 and IL-10 have been reported to peak within the first 6 hours of islet infusion and gradually subside to pretransplant levels days from islet transplantation [16–18]. To demonstrate this further, we recently analyzed a marker of beta cell stress: the insulin-to-proinsulin index ratio, in subjects pre- and post-TPAIT. The latter ratio has been a well-established marker of beta cell stress in the allotransplantation setting [21, 22]. The impaired processing of the precursor proinsulin to insulin, and the subsequent lowering of insulin-to-proinsulin index ratio is characteristic of impaired allogenic islet cell function, and has been reportedly present in early allograft failure [18, 21]. In our autologous islet transplantation analysis, three years post-TPAIT, we observed a significantly lower insulin-to-proinsulin index ratio in insulin-dependent subjects compared to those fully insulin independent [23]. Intriguingly, a significantly lower insulin-to-proinsulin index ratio was also seen in insulin-independent subjects post-TPAIT when compared to their pre-TPAIT state [23]. Stemming from the detrimental effects of inflammation peri-transplant on the decline of beta cell mass, rationally-selected anti-inflammatory approaches for patients undergoing TPAIT are increasingly investigated [24, 25]. Such approaches may protect islets from the initial inflammatory challenge; resulting in preservation of beta cell function and leading to sustained long-term insulin independence.

Additionally, for maximization of islet mass infused with concomitant avoidance of increased portal pressure and/or obstruction of portal flow, several groups have looked into alternative, extrahepatic sites for islet transplantation. This approach is further supported by the need to mitigate hypoglycemia associated with intrahepatic infusions. The increased risk of hypoglycemia with exercise and post-meal intervals in subjects with strict intrahepatic infusions of islets has been hypothesized to be due to inhibition of secretion of glucagon by islets surrounded and bathed in the abundant intrahepatic free glucose environment [26]. Additionally, intrahepatic islets are classically exposed to higher concentrations of toxins and/or immunosuppressive agents, all of which can accelerate deterioration of beta cell function. Finally, recent evidence suggests that the latter liver-related factors might be the confounding factor in the relationship between islet mass yield and metabolic outcomes posttransplantation, thereby mandating more research on extra-hepatic sites of transplant [27]. Currently, several centers are utilizing the peritoneal cavity for infusion of excess, remaining islets after liver infusion [28]. Attempts at delivery of the whole islet cell mass in non-hepatic sites, such as the omentum, peritoneal cavity, bone marrow and intramuscular sites are well-underway [29, 30].

## 3. Allogenic Islet Transplantation

Islet allotransplantation entails the isolation of islets from donated pancreases of deceased donors without diabetes, and subsequent intrahepatic engraftment in recipients via the portal vein. Islet allotransplantation is reserved for type 1 diabetes mellitus subjects with extreme glycemic variability and/or severe hypoglycemia complicated by hypoglycemia unawareness, or in subjects with functional renal graft. Compared to the more invasive and costly procedure of whole-organ pancreatic transplantation, islet transplantation offers a less aggressive and more affordable route for restoration of beta cell function [1, 2].

Metabolic outcomes of allogenic islet transplantation differ across recipients. In its ninth report, the Collaborative Islet Transplant Registry (CITR) published outcomes of 1,011 islet allograft recipients, transplanted between 1999 and 2013 [31]. The registry included 819 allogenic islet transplants alone and 192 islet transplants post-kidney engraftments. The report concluded 50% of adults with type 1 diabetes achieved insulin independence one-year post-transplantation (alone or post-kidney). Rates of insulin independence, however, declined with time. At 5-years post-transplantation, 30% of islet transplants alone were insulin independent compared to only 20% of islets post-kidney [31].

With improvements in procurement procedures, along with the development of less toxic but more effective immunosuppressive agents, the success rates of allogenic islet transplantation have significantly improved. Rates of insulin independence reportedly increased from 27% to 37% to 44% in the eras between 1999-2002, 2003-2006 and 2007-2019, respectively [32]. The need to receive subsequent, additional islet infusions also reduced significantly from 60% to 65% to <50% in the most recent period [25]. Despite the majority (>90%) reported to have severe hypoglycemic episodes pretransplant, over 90% were hypoglycemia-free post-transplant, and up to 5 years of follow up [32]. Additionally, 60% of subjects were able to achieve near-normal glycemic control (HbA1c <6.5%) with the first 3-5 years of follow up [32].

Metabolic outcomes of allogenic islet transplantation have also been contrasted against intensification of insulin therapy via continuous insulin infusions, of which the former was found to be superior in several metabolic and glycemic aspects. In the recent multicenter, randomized controlled trial of islet transplantation versus insulin therapy in patients with type 1 diabetes with severe hypoglycemia or poorly controlled glycaemia after kidney transplantation (TRIMECO), islet transplantation was shown to produce significantly lower HbA1c (5.6% vs 8.2%), superior protection from severe hypoglycemia, and significantly improved quality of life compared to intensive insulin therapy, at 6 months post-transplantation [33].

A key factor associated with a higher likelihood of insulin independence post-allogenic islet transplantation is the volume of islets transplanted, as with autologous islet transplantation. In the allogenic setting, this often translates to procurement of islets from multiple deceased donors for maximization of yield. With islets making up less than 2% of the mass of an adult pancreas, procurement of healthy islets from multiple deceased donors has proven challenging. Attempts at identifying other sources of viable islets has been the subject of many ongoing researches. A major field of interest has been human embryonic stem cells [34–37]. Attempts at differentiating stem cells in vitro to endodermal lines, with further differentiation to glucose-sensing, insulin-producing cells in mice have proven successful [34, 36]. Current efforts are directed at inducing such differentiation to beta-like cells in vitro, for enabling of large volume-production of islets made for human transplantation purposes [37].

Another explored source for islet cell development and production has been xenoislet transplantation. In animal studies, intrahepatically transplanted porcine islets have been shown to maintain normoglycemia in autograft models, suggesting a lesser predisposition to metabolic stress overtime [38]. A major drawback of xenoislet transplantation has been the increased risk of hyperacute rejection with porcine immunological triggers, thereby mandating more aggressive immunosuppression regimens [39, 40]. Current novel approaches are targeting the porcine islet genome, possibly creating a resource for porcine islets less immunogenic for human transplantation purposes than the current form [41].

As with autologous transplantation, inflammation escalating at the time of allotransplant has been shown to lower the functional islet cell mass, estimated at a loss of up to 25% of total islet yield [42]. One of the main toxic cytokines implicated in toxicity of beta cells at time of infusion has been TNF-α [43]. Current approaches adapted incorporate the slow-release pentoxifylline agent, an inhibitor of TNF-α production, to be started 2 days prior to transplant and continued for 7 days post-transplant. Additionally, and immediately prior to operative islet infusion, the TNF-α inhibitors etanercept is intravenously administered, and continued on days 3, 7 and 10 post-transplantation [43].

## 4. Conclusions

Pancreatic islet transplantation is a rapidly evolving field. Efforts are currently focused on improving islet quality and yield, in the autologous and allogenic setting, for improvement of metabolic outcomes and quality of life in the long-run.
